# Incidence, clinical features, and outcomes of COVID-19 in Canada: impact of sex and age

**DOI:** 10.1186/s13048-020-00734-4

**Published:** 2020-11-24

**Authors:** Jacob O’Brien, Kevin Y. Du, Chun Peng

**Affiliations:** 1 Department of Biology, York University, Toronto, Canada; 2 Centre for Research in Biomolecular Interactions, York University, Toronto, ON Canada

**Keywords:** COVID-19; SARS-CoV2; sex, Age, Canada, Estrogens, Incidence, Symptoms, Fatality

## Abstract

Male sex and older age have been reported to be associated with worse outcomes from COVID-19. It was postulated that estrogens may play a role in reducing the severity of the disease and may therefore offer a treatment option for COVID-19 patients. However, more female cases and deaths from COVID-19 have been recorded in Canada. To determine the potential role of estrogens, we analyzed COVID-19 data from Canada, focusing on the impact of sex and age. Although the overall incidence rate is higher in females than in males, when several high risk groups, including health care workers and long-term care residences, which are predominantly females, were excluded, we found that females had a lower incidence rate than males between the ages of 20s to 70s. Interestingly, this sex-based difference is more evident in females of the reproductive ages (20–49) than in postmenopausal patients (60s or older). Males have significantly higher hospitalization, ICU admission, and case fatality rates; however, a greater difference was observed in the older age groups. Finally, symptom manifestation varied between sexes. Some of the symptoms, which were more frequently observed in patients who recovered than patients who died, were more commonly observed in females of the reproductive age compared to their male counterparts. Since only females of the reproductive age have much higher circulating estrogens than males, these findings suggest that estrogens may play a role in reducing COVID-19 incidence and in the development of symptoms, especially those related to better survival.

## Introduction

As of September 20, 2020, over 30.6 million people in the world have been diagnosed with coronavirus disease-19 (COVID-19), caused by Severe Acute Respiratory Syndrome Coronavirus-2 (SARS-CoV-2) infection, and nearly 1 million have died from this disease worldwide [1]. SARS-CoV-2, originally termed 2019 novel coronavirus (2019-nCoV), was first discovered in Wuhan, China in December 2019 [2], has now spread to all continents in the world, eliciting an unprecedented global health crisis. Age and sex have been identified as two of the prominent risk factors in COVID-19 deaths [3].

Early epidemiological studies conducted in China, India, and Iran revealed that fewer females were infected by SARS-CoV2 [2, 4–9]. These results suggest that females may be less susceptible to SARS-CoV-2 infection and/or less likely to present symptoms of COVID-19. However, with the rapid spread of SARS-CoV-2 in the world and an increase in epidemiological studies around the globe, more recent studies found that there were no significant differences between men and women in the incidence of COVID-19 [10, 11]. On the other hand, many studies have reported that female patients have better outcomes than male patients. Analyses of COVID-19 cases in China showed that females accounted for a higher number of mild cases and fewer deaths [12], with the ratio of males to females who died from COVID-19 as high as 2.4 [13]. Similar findings were reported for several European countries, including France, Italy, Germany, Spain, and Switzerland, where males had more than 50% higher rates in hospitalization and death due to COVID-19 [14]. In New York City, male COVID-19 patients had a higher hospitalization and death rate than females [5, 6]. The Global Health 50/50 has tracked sex-disaggregated COVID-19 data from 47 countries and found that in most countries, women have a lower COVID-19 mortality rate than men [10].

Although the mechanisms underlying the sex-specific COVID-19 outcomes are not entirely clear, it is possible that this involves a complex interplay among biological, behavioural, environmental, and socioeconomic factors. Sex differences in the immune response to infectious diseases and the role of sex steroids regulating immunity have been reported [15]. The production of sex steroids fluctuates with age; women of reproductive age produce significantly more estrogens than men, prepubertal girls, and postmenopausal women. It has been proposed that estrogens may exert protective effects against COVID-19 [14, 16] and a clinical trial is underway to determine if estradiol can reduce the severity of COVID-19 infection (https://clinicaltrials.gov/ct2/show/NCT04359329). If estrogens play a key role in the sex-based responses to COVID-19, there would be a greater sex-based difference in outcomes when females are reproductively active. However, to the best of our knowledge, no detailed studies have been reported to compare sex differences in COVID-19 incidence, clinical characteristics, and outcomes between women of reproductive and non-reproductive ages.

The COVID-19 data reported by health agencies in Canada show that more females were diagnosed and died from it. This is at odds with findings from most other countries. We therefore analysed the characteristics and outcomes of COVID-19 cases in Canada, focusing on the potential impact of sex and age on the incidence, symptoms, hospitalization/ICU rate, as well as fatality. Our findings suggest that women have a lower risk of contracting COVID-19 and estrogens may play a role in protecting females from being infected by SARS-CoV-2. Interestingly, we also found that there is a significant difference in COVID-19 symptoms between males and females, especially in the reproductive age group.

## Materials and methods

### Datasets

All Canadian COVID-19 case data was acquired from the July 27, 2020 Statistics Canada preliminary dataset (Table 13-10-0781-01) [17]. In collaboration with the Public Health Agency of Canada, Statistics Canada was continuously collecting nation-wide case information of laboratory diagnosed cases of COVID-19 from all provincial and territorial health ministries. The data was considered preliminary as new cases were being added as they were diagnosed, although this was not in real time, there was a lag between diagnosis and collation. The most recent Canadian population demographic estimate from 2019 was used for normalization to cases per 100,000, where applicable [18]. To normalize health care worker cases, the 2016 census for Canadian workforce demographics was used [19]. Dataset preparation is discussed in more detail within the supplemental materials and methods.

**Fig. 1:**
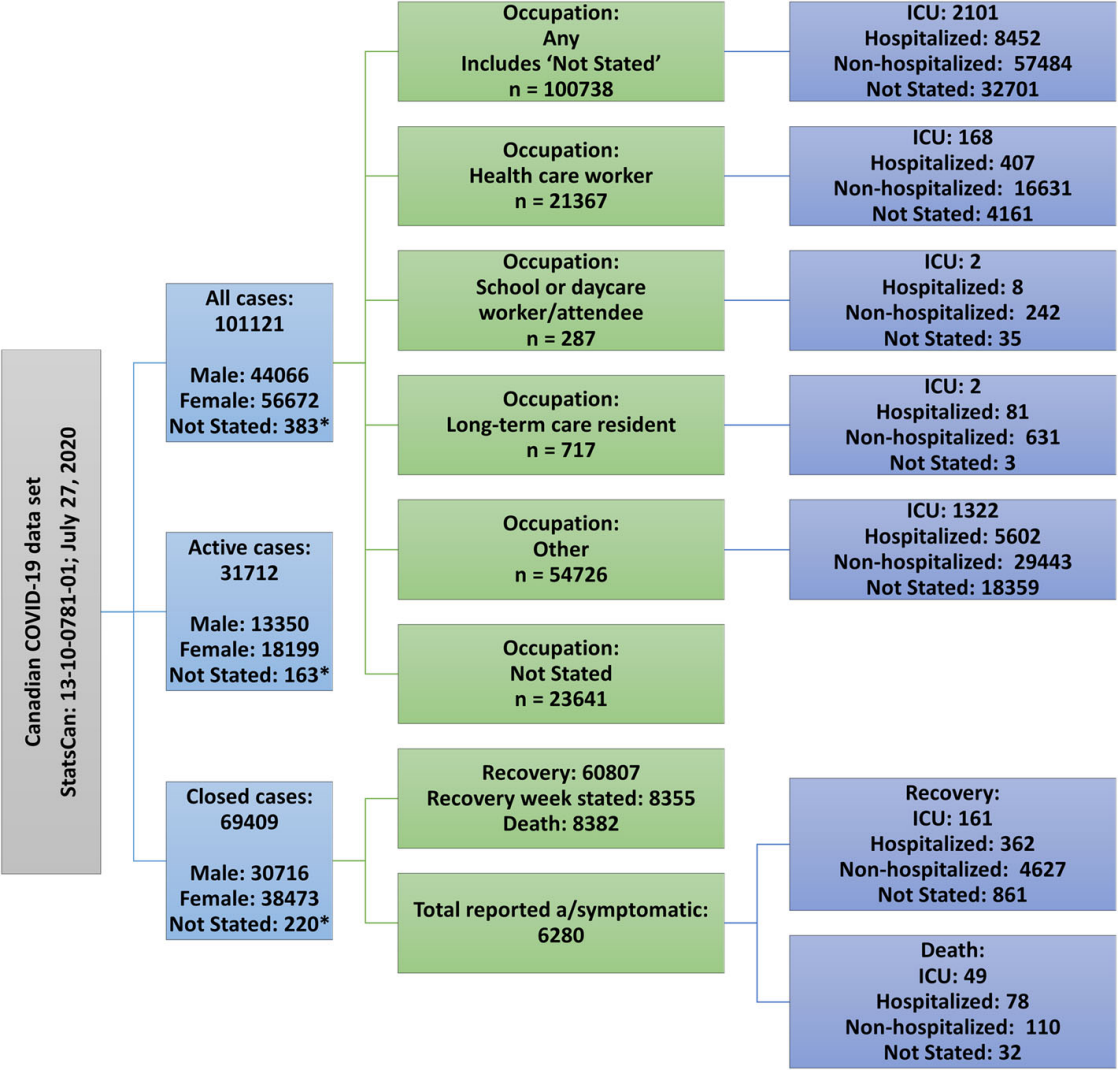
Overview of the Public Health Agency of Canada’s preliminary Canadian COVID-19 dataset. Cases were divided into three main groups: All, Active, and Closed cases. Closed cases are those with a stated recovery or death. Cases where the sex was Not Stated* were excluded from analysis, and unless explicitly stated, cases where the parameter of interest was Not Stated were also excluded. Symptomatic cases had at least one reported symptom whereas asymptomatic cases either reported Asymptomatic or reported ‘No’ to all symptoms. Hospitalization status was divided into four groups: ICU, Hospitalized but not in ICU, Non-hospitalized, and Not Stated

The preliminary COVID-19 dataset was divided into three groups: All Cases, Active Cases, and Closed Cases (Fig. 1). As of July 27, 2020, 101,121 total cases were reported. Of those cases, 69,409 were closed due to either recovery or death. Depending on the analysis, either All Cases or Closed Cases were used; cases where sex was not stated were excluded. The All Cases group was also disaggregated by occupation and hospitalization status. Analyses of death, recovery, or symptom incidence used the Closed Cases group. A case was a/symptomatic if at least one symptom was reported or all symptoms were reported ‘No’ or the case reported asymptomatic. See Suppl. Fig. 2 for a list of parameters available in the COVID-19 dataset.

**Fig. 2:**
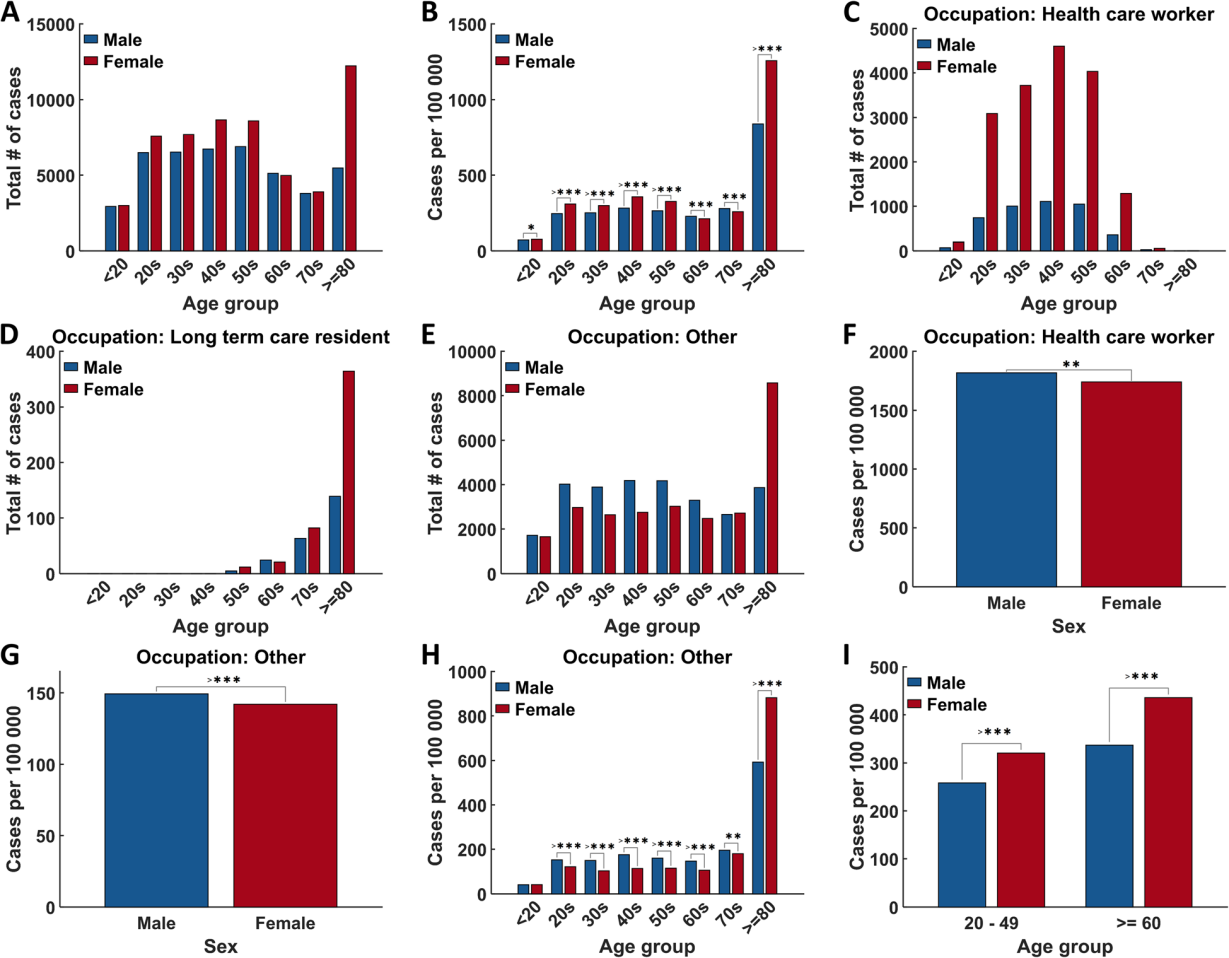
Age, sex, and occupation disaggregated COVID-19 incidence rates. **A)** Age/sex distribution of patients from ‘All cases’. **B)** Age/sex distribution of patients from ‘All cases’ normalized to population demographics. **C-E)** Age distribution of patients with occupation. Occupation “Other” refers to all occupations except health care workers, long-term care residences, and school/daycare workers/attendees. **F)** Sex disaggregated Health care worker cases normalized to workforce population demographics (any degree/certificate, all ages, male/female, ‘3 Health occupations’). **G, H)** Sex and/or age disaggregated ‘Occupation: Other’ cases normalized to Canadian population demographics. **I, J)** Age disaggregated into female reproductive age (20–49) and postmenopausal age (> = 60) for ‘All cases’ (I) and ‘Occupation: Other’ cases (J). All data was sampled from the ‘All cases’ group. Demographic normalized data was analyzed using *X*^*2*^ goodness-of-fit. *p < 0.05, **p < 0.01, ***p < 0.001, >***p < 0.0001

### Statistical analysis, disaggregation, and normalization

For means with one or two groups, either one- or two-way ANOVA was used, followed by Tukey’s multiple comparisons. For nominal (categorical) counts, either *X*^*2*^ goodness-of-fit or Fisher’s Exact Test was used. A *p*-value < 0.05 was considered significantly different. The exact test used is reported within the figure legend of each figure.

For age and sex disaggregation, the set of cases was first selected, e.g. All Cases, Closed Cases, etc. (Fig. 1). Analyses investigating death/recovery or a/symptomatic cases used Closed Cases. After set selection, cases were segmented into age groups of less than 20 years old, 20–29, 30–39, 40–49, 50–59, 60–69, 70–79, and 80 and greater and then separated into male or female cases. For reproductive age groups, cases meeting the criteria were segmented into either 20–49 or > = 60 years of age.

The data were then reported as absolute values (‘Total # of cases’), population normalized values (‘Cases per 100 000’, ‘Deaths per 100 000’), or as a percentage of cases within each group. To calculate normalized values, the 2019 Canadian Population estimate was used. The number of male and female Canadians for each age group was summed and the absolute value of cases for the corresponding age/sex group was divided by the population in that group and multiplied by 100,000. Hospitalization and ICU admission rate was calculated as a percentage of the number of cases hospitalized and admitted to ICU, respectively, to the total number of cases in that group.

To normalize Health Care Worker cases, the Canadian Workforce demographic survey was used. The process was identical to population normalization except the total number of male and female ‘Health Occupation’ workers were used. The age demographic groups of the Workforce survey did not align with the COVID-19 dataset and as a consequence, we were limited to disaggregation into only male or female cases.

Analysis of symptoms incidence was slightly more complicated as some symptoms were reported as ‘Not Stated’ more often than others. Because of this, each symptom was analysed independently of the others. The closed cases that satisfied age, sex, and/or other criteria were first selected and then only cases that reported ‘Yes’ or ‘No’ to the test symptom or asymptomatic were retained for analysis. Incidence of symptoms was expressed as the percentage of the number of cases that reported “Yes” to a specific symptom relative to the total number of the cases within each group. All analyses were conducted in MATLAB R2020a Update 4.

## Results

### Females have a lower incidence rate

We first compared the total number of cases among males and females of different age groups and found that there were more female patients between ages 20–50 and above 80 (Fig. 2A). We assumed these cases were effectively randomly sampled from the population. By normalizing these cases to the Canadian population demographics, we were able to make comparisons between age and sex groups. When expressed as the number of cases per 100,000 people, the same statistically significant trend was observed (Fig. 2B). Since all provinces and territories in Canada, to varying degrees, have restricted travels, prohibited large gatherings, closed schools and non-essential businesses, we reasoned that essential workers would have a much greater chance of being infected by SARS-CoV-2 than the general public. In addition, there were outbreaks reported in many long-term care and some daycare facilities. We therefore disaggregated health care workers and long-term care residence cases and compared the incidence rate between males and females of different age groups. For health care workers, there was a much higher total number of cases for females in all age groups (Fig. 2C). Among long-term care residents, more female patients, especially in the 70 and 80 age groups, were noted (Fig. 2D). When these groups were excluded, females had lower incident rates in the age groups of 20s to 60s (Fig. 2E). When the case numbers were normalized to the occupation demographics, female health care workers had a lower number of cases per 100,000 than their male counterparts (Fig. 2F). When health care workers, long-term care residents, and school/daycare workers/attendees were excluded, there was a lower number of female patients than male patients (Fig. 2G). Interestingly, females had a lower incidence rate only in the age groups ranging from 20s to 70s, while there were significantly more female than male patients in the age group of 80s and older (Fig. 2H). To determine if estrogens may play a role in protecting females from COVID-19, we selected two age groups of women, one included women of the reproductive age (20–49) and the other with postmenopausal females (60 or older). The age group of 50–59 was excluded as this group is likely to have both pre- and postmenopausal women. Interestingly, we observed a significant decrease in the incidence rate among females of the reproductive age when compared with males of the same ages; however, in the older age group, females had a higher incidence rate than males (Fig. 2I).

### Females have lower hospitalization, ICU admission, and case fatality rates

**Fig. 3:**
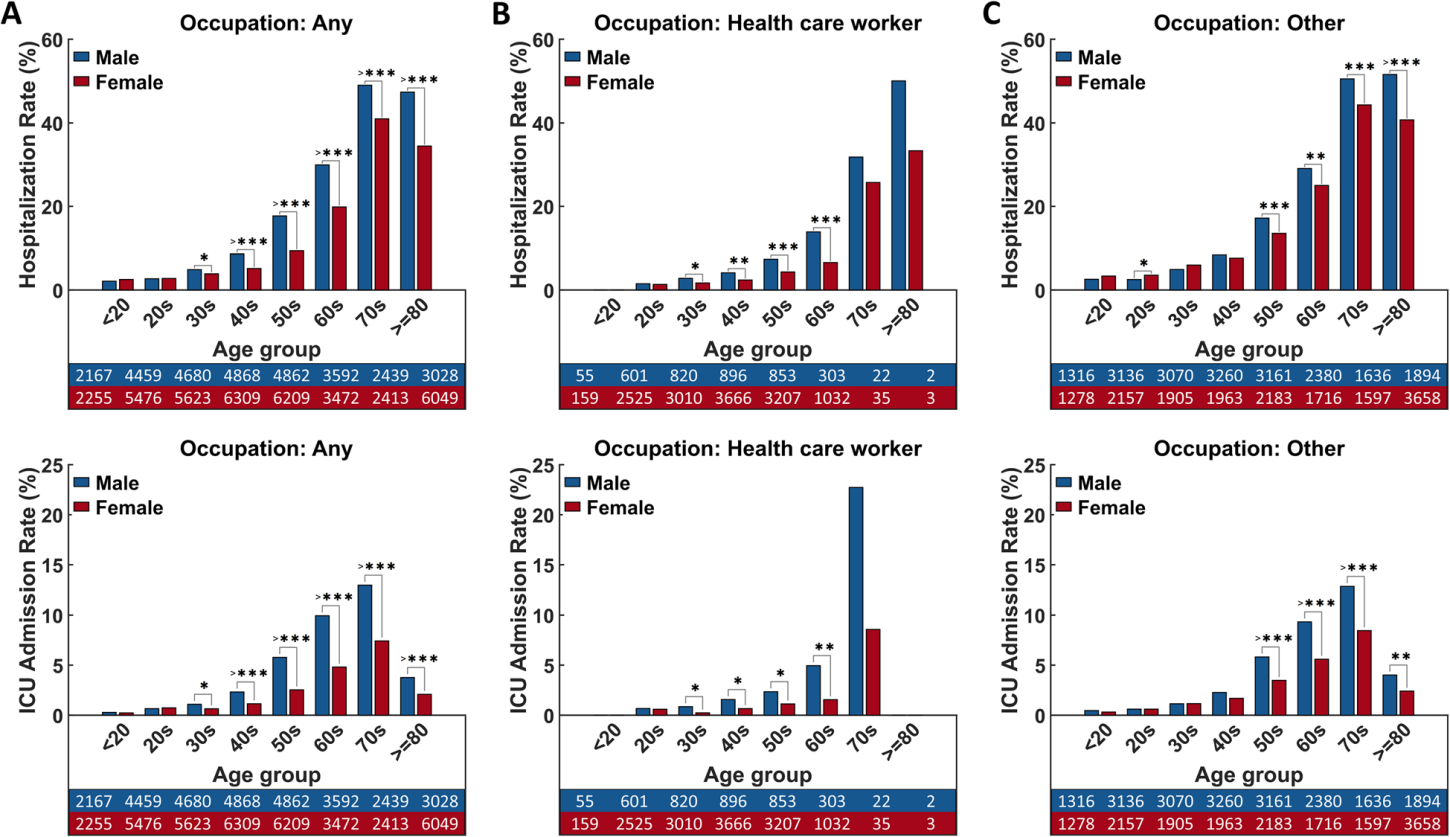
Effects of sex and age on hospitalization and ICU admission. A) Hospitalization and ICU admission rate among all patients. B) Hospitalization and ICU admission rate among health care workers. C) Hospitalization and ICU admission rates among other occupations. Data are represented as percentages of cases either hospitalized (including ICU patients; top row) or in ICU (bottom row) relative to the total number of cases per sex in each age group. The tables represent the total number of cases with a reported hospitalization status and occupation, ordered by age group (left to right) and sex (male top, female bottom). Data were sampled from ‘All cases’ group and analyzed with Fisher’s Exact Test. *p < 0.05, **p < 0.01, ***p < 0.001, >***p < 0.0001

**Fig. 4:**
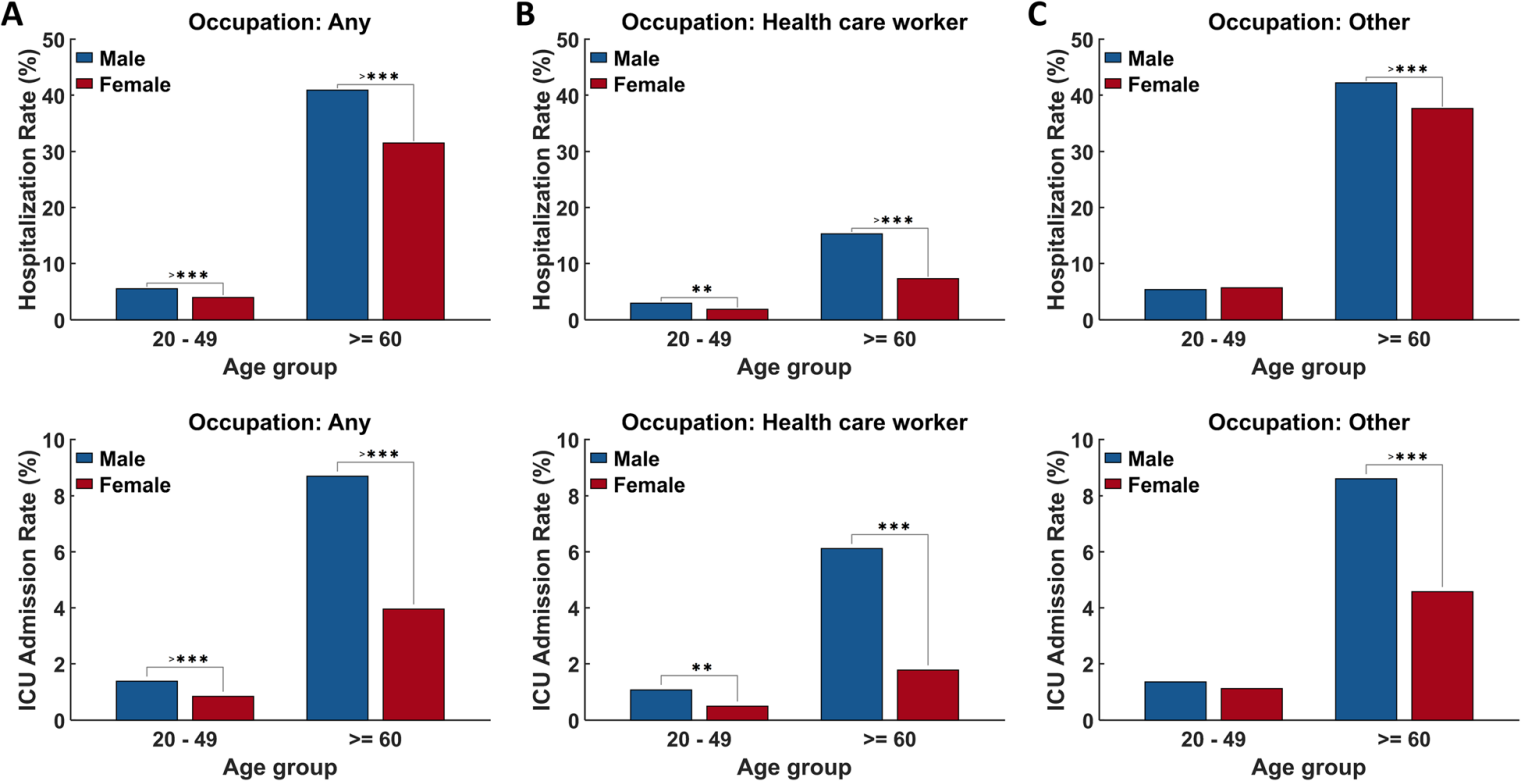
Reproductive age, sex, and hospitalization status disaggregated COVID-19 cases. **A)** Any occupation, **B)** Health care workers, **C)** Other occupations. Similar analyses were performed as in Fig. 3 except that the age groups 20s, 30s and 40s were combined into the group of 20–49 to represent females of reproductive ages and the age groups of 60s, 70s, and > 80s were combined to represent postmenopausal females and their age-matched males. *p < 0.05, **p < 0.01, ***p < 0.001, >***p < 0.0001

We compared females and males in different age groups for the rate of hospitalization, ICU admission, recovery time, and case fatality. When all cases were considered, there was a significantly lower number of hospitalization and ICU admission in females aged 30 or older (Fig. 3A). Similar findings were observed in health care workers with fewer females being admitted to hospitals and ICUs (Fig. 3B). When health care workers, long-term care residents, and school/daycare workers/attendees were excluded, a significant reduction of female cases in hospitalization and ICU admission was only observed in the age groups of 50 or higher (Fig. 3C). Again, we compared sex differences in hospitalization and ICU rates between 20 and 49 and 60 and older age groups. Surprisingly, we found a more significant reduction in hospital and ICU admission rates in the older age group compared to the reproductive age group (Fig. 4).

**Fig. 5:**
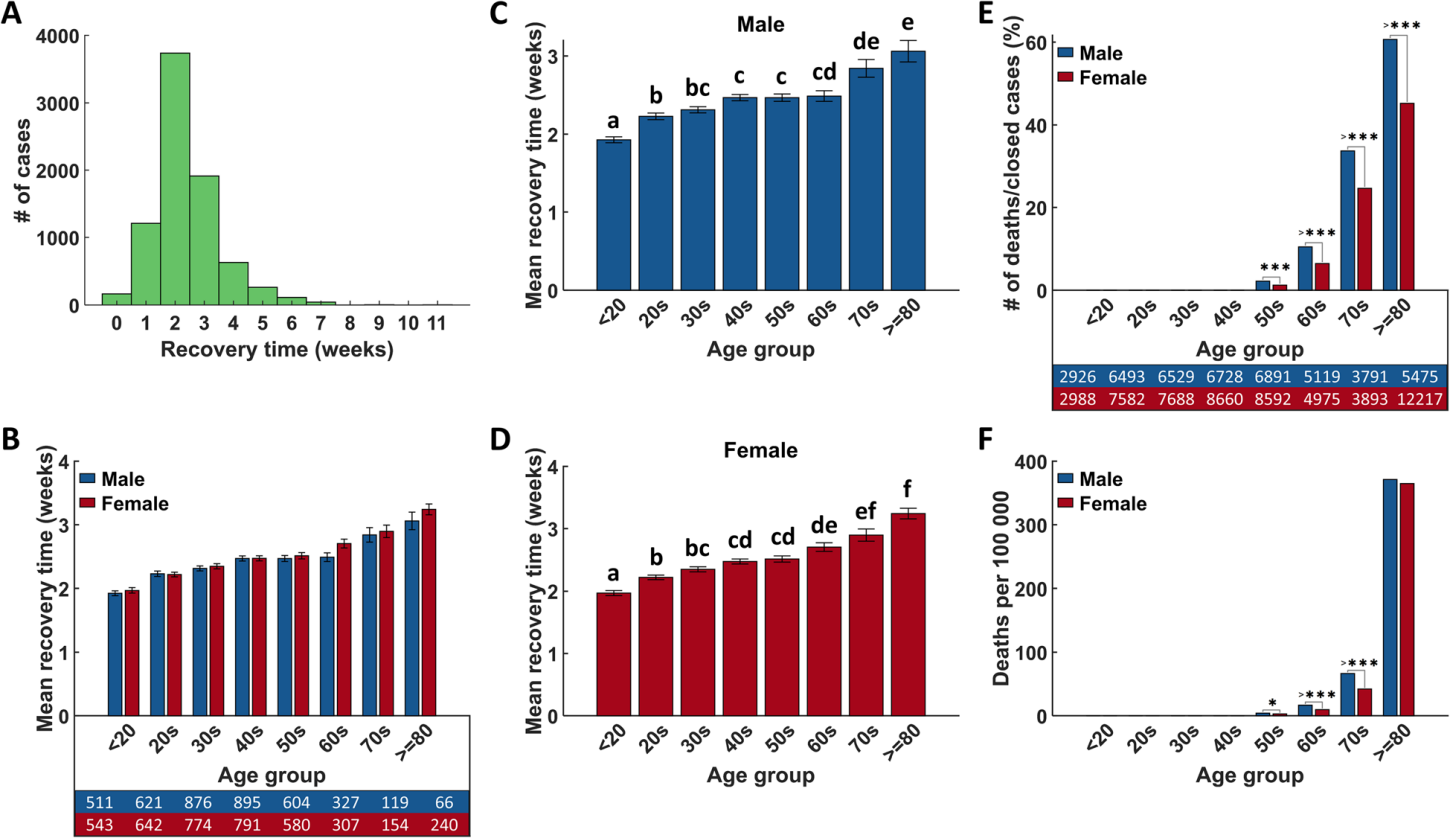
Recovery and death statistics of age and sex disaggregated COVID-19 cases. **A)** Histogram of recovery time. **B-D)** Sex/age disaggregated recovery time analyzed with one- or two-way ANOVA followed by Tukey’s multiple comparisons. Different letters above bars denote statistical significance (p < 0.05). Data represent mean ± SEM. **E)** Percentage of deaths relative to total number of closed cases per sex in each age group analysed with Fisher’s Exact Test. **F)** Death incidence normalized to Canadian population and analysed with *X*^*2*^ test. The tables represent the total number of cases with a recovered (B) or survival outcome (E) ordered by age group (left to right) and sex (male top, female bottom). All data were sampled from ‘Closed cases’. *p < 0.05, ***p < 0.001, >***p < 0.0001

The majority of patients took 1–4 weeks to recover (Fig. 5A). There was no significant difference in the recovery time between males and females in any age groups (Fig. 5B). However, in both sexes, older patients took longer to recover (Fig. 5C, D). All deaths occurred in patients 50 or older. A significant decrease in case fatality was observed in females compared with males of the same age group (Fig. 5E). Due to zero reported death in the younger age groups, we were unable to compare the case fatality rate between sexes in the 20–49 and 60 or older age groups. When normalized to the Canadian population demographics, the number of deaths in patients 80 or older was nearly 4 times more per 100,000 than patients only 10 years younger and only females younger than 80 had a significantly lower death rate than males of the same age group (Fig. 5F).

### Sex differences in clinical manifestation of COVID-19

Overall, 83.6% of the total reported a/symptomatic cases were symptomatic. Among these symptomatic patients, more females recovered and more males died. On the other hand, more males that recovered were asymptomatic compared to recovered females. It is interesting to note that death also occurred in asymptomatic patients and at the same proportion in females and males (Table 1). The four most common symptoms of COVID-19 in both male and female patients with no difference in rates were cough, chills, weakness, and pain. Fever was reported by more male than female patients. On the other hand, more females experienced a sore throat, runny nose, shortness of breath, nausea, and headache, as well as diarrhea (Table 2).

**Table 1:**
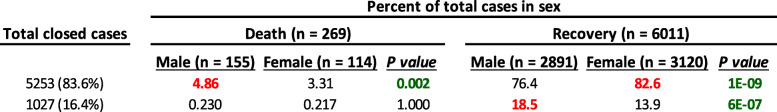
Total closed symptomatic and asymptomatic cases. Data represent the proportion of either male or female cases in each category relative to total male or female cases. *P* values highlighted green were considered statistically significant and values highlighted red were the larger value in the comparison. Data were from the ‘A/Symptomatic, Closed cases’ group and analyzed with Fisher’s Exact Test

**Table 2:**
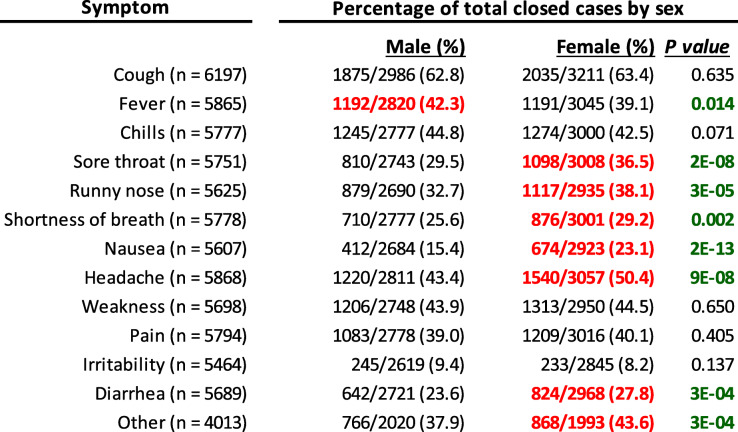
Overall incidence of COVID-19 symptoms in males and females. Data represent the proportion of cases with each symptom relative to the total number of cases where the symptom is reported ‘Yes’ or ‘No’ or the case reported asymptomatic. P values highlighted green were considered statistically significant and values highlighted red were the larger value in the comparison. Data were from the ‘A/Symptomatic, Closed cases’ group and analyzed with Fisher’s Exact Test

**Fig. 6:**
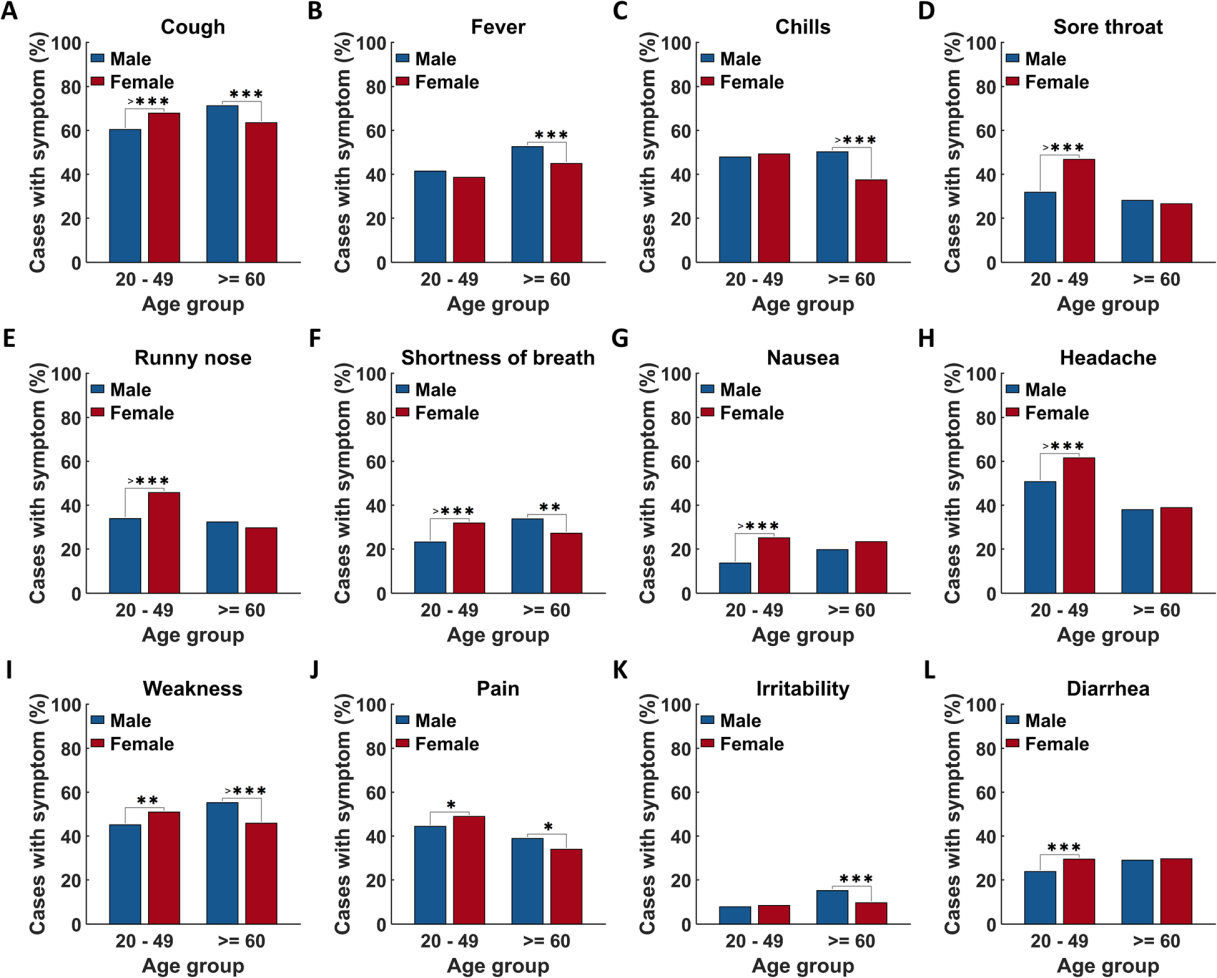
Reproductive age, sex, and symptom disaggregated COVID-19 cases. Data represent percentage of cases with symptom relative to total closed cases per sex in each age group. Data were sampled from the ‘A/Symptomatic, Closed cases’ group and analyzed with Fisher’s Exact Test. *p < 0.05, **p < 0.01, ***p < 0.001, >***p < 0.0001

We then determined differences between male and female patients in symptoms more likely to associate with death or recovery (Table 3). We found that 72.8% of males who died from COVID-19 had a cough, which is significantly higher than males who recovered from the disease or females that died (Table 3, lower). More patients who had a fever, shortness of breath, and irritability died, regardless of sex, while cough and weakness were also associated with death in males. In both sexes, patients who recovered had a higher chance of developing chills, sore throat, runny nose, headache, and pain than patients who died and these symptoms, excluding chills and pain, were more frequent in recovered females than males (Table 3, upper). Interestingly, these symptoms were more frequently reported by female patients of the reproductive age (Fig. 6 and Fig. S3). These symptoms were also reported at a significantly higher rate by women than men among non-hospitalized cases (Table 4).

**Table 3:**
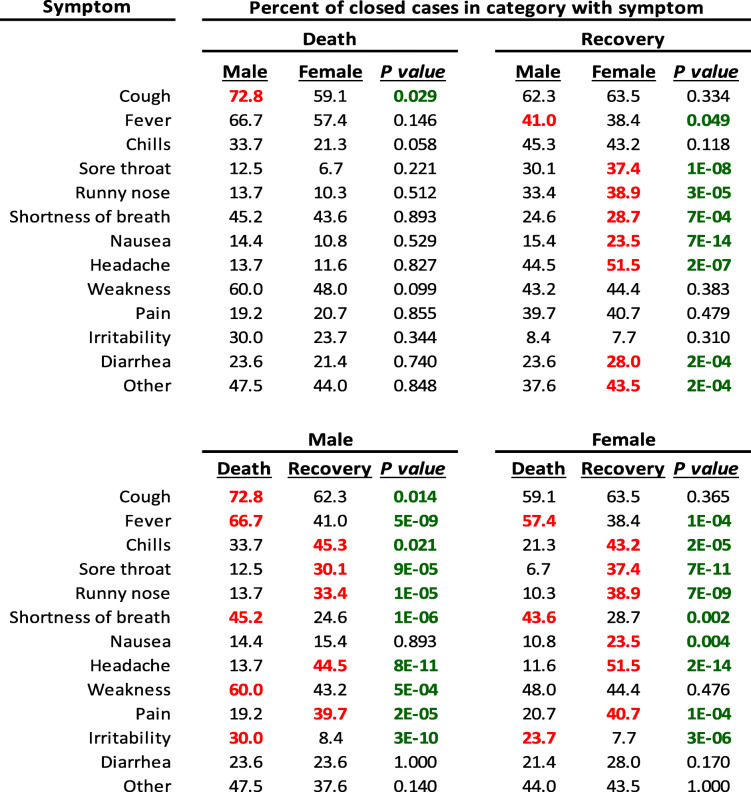
Overall proportions of symptoms in survivors and non-survivors. Data represent the proportion of cases per category that had that symptom. The upper table compares symptom incidence between male and female cases that died or between cases that recovered. For example, males who died reported cough more often than females who died. The lower table compares within males or females, between cases that died or recovered. For example, headache was reported more often in females who recovered than females who died. P values highlighted green were considered statistically significant and values highlighted red were the larger value in the comparison. Data were from the ‘A/Symptomatic, Closed cases’ group and analyzed with Fisher’s Exact Test

**Table 4:**
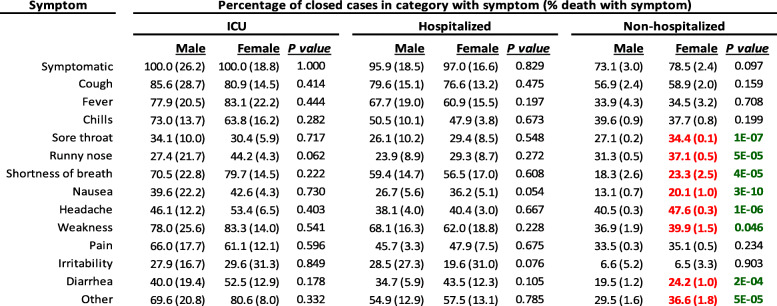
Incidence of symptoms in males and females based on hospitalization status. Data represent the proportion of cases per category that had that symptom. The proportion of cases that die having that sympmtom, in its category, are in parentheses. For example, a higher proportion of non-hospitalized females vs. males reported sore throat. There were no statistical differences in death proportions between males and females when comparing within each hospitialization status, e.g. the proportion of males or females that died in the ICU with cough were not significantly different. P values highlighted green were considered statistically significant and values highlighted red were the larger value in the comparison. Data were from the ‘A/Symptomatic, Closed cases’ group and analyzed with Fisher’s Exact Test

## Discussion

In this study, we show that although the absolute number of COVID-19 cases was higher for females, females have a lower COVID-19 incidence rate when high-risk populations, namely health care workers and long-term care residences, were disaggregated. These two groups, which are predominantly females, were at a much higher risk of contracting SARS-CoV-2 likely due to higher exposure rates in hospitals and outbreaks in long-term care facilities. However, we also found that women at the age of 80 or older had a higher chance of developing a diagnosable case of COVID-19. Our finding that overall, females have a lower incidence of COVID-19 is consistent with early reports from several countries [2, 4–9] and likely explains why later data show no clear difference in COVID-19 incidence between sexes. Since many countries/regions applied state or regional lockdowns, many new cases are essential workers, such as health care professionals, who are predominantly females. It is very likely that if men and women had a similar chance of SARS-CoV-2 exposure, there would be fewer cases for female patients younger than 80 years old.

After puberty and before menopause, women have much higher circulating estrogens, especially estradiol, than males and prepubertal and postmenopausal females. In this study, we found that females had a lower COVID-19 incidence rate than males unless they were 80 or older. Interestingly, when comparing the age groups of 20–49 and 60 or older, we found only females of the reproductive age group had a lower incidence rate than males. This result suggests that estrogens may exert protective effects in reducing COVID-19 infection. However, females from 60s to 70s still have lower incidence rates than males. Since there are no major differences in estrogen levels between sexes after menopause, it is highly probable that additional factors are also important in determining susceptibility to COVID-19.

One of the possible mechanisms by which estrogens reduce COVID-19 incidence is to inhibit SARS-CoV-2 entry into host cells. Angiotensin-converting enzyme II (ACE2) and transmembrane protease serine 2 (TMPRSS2) are located on the cell membrane and mediate the entry of SARS-CoV-2 into cells [20, 21]. Using normal human bronchial epithelial cells from a single female donor, it was reported that treatment with estradiol significantly reduced the mRNA levels of ACE2, but not TMPRSS2 [22], suggesting that estrogens may inhibit the entry of SARS-CoV2 into cells. However, it has also been reported that ACE2 may have protective effects against SARS-induced lung damage [23]. A recent analysis has shown higher ACE2 levels in Asian women and younger age groups but lower levels in men and older age groups [24]. In an unpublished study, Wang et al. (2020) used computational approaches to analyze RNA-seq datasets that involved drug treatments and identified estrogenic and androgenic compounds as transcriptional modulators of TMPRSS2. Specifically, they found that estradiol and a phytoestrogen, genistein, reduced TMPRSS2 mRNA levels. In contrast, testosterone and synthetic androgens induced the expression of TMPRSS2 [25]. However, a recent study found the expression of TMPRSS2 mRNA in lung tissues was not significantly different between males and females [26]. Thus, whether or not estrogens regulate ACE2 and TMPRSS2 to reduce SARS-CoV-2 infection requires further investigation.

A recent study comprehensively compared differences in immune responses between male and female COVID-19 patients and found that males had higher circulating innate inflammatory cytokines, interleukin (IL)-8 and IL-18, and stronger induction of non-classical monocytes while females had a more robust T cell activation and higher interferon (INF)α2 [27]. These differences in immune response may explain the different COVID-19 incidence and clinical outcomes between sexes. Although the mechanisms underlying the sex-differences in immune responses are not well understood, sex hormone receptors, notably the estrogen and androgen receptors (ER and AR respectively), are widely expressed in immune cells and play a role in regulating immunity [28]. Estrogens have been reported to have immunostimulatory or immunosuppressive effects depending on concentration and cell types, while testosterone is immunosuppressive [29]. Females may elicit stronger innate immune responses to viral infections due to greater production of type I interferons (IFNs), which induce anti-viral effects and type 1 immunity [29]. Type I IFNs are released by plasmacytoid dendritic cells through stimulation of toll-like receptors (TLR), specifically in coronaviruses by recognition of single-stranded RNA by TLR7 [30]. IFN-α release was significantly higher in female than male lymphocytes upon TLR7 stimulation [31]. Estrogens may contribute to this phenomenon, as estradiol promotes TLR-activated IFNα release through ERα [30].

Similar to many previous studies [14, 32, 33], we also found that female patients have lower hospitalization, ICU admission, and case fatality rates than males. However, to our surprise, the sex-based differences were greater in the older age groups than in the reproductive age group. Although this could be due to the low rate of hospitalization and death in younger patients, the fact that this sexual difference is greater in postmenopausal women suggests that other factors, rather than estrogens, are more important in reducing the severity of COVID-19. Several genes on the X-chromosome are known to regulate immune response and it has been suggested that females have a lower viral load and inflammation [34]**.** In addition, sex differences in chronic diseases are also commonly observed. Some of these diseases, such as diabetes and cardiovascular disease, are risk factors for poor COVID-19 outcomes [3, 33, 35, 36]. According to the Public Health Agency of Canada (PHAC), the prevalence of diabetes and heart diseases are higher in males than in females, especially in the age groups of 45 and older [37, 38]. These conditions may explain the higher ICU and hospitalization rates in males, particularly in the older age populations.

One of the interesting findings from this study is the differences in symptom presentation between males and females. Analyses of sex disaggregated data of symptoms show a higher rate of fever in males, while several other symptoms, such as sore throat, runny nose, nausea, diarrhea, shortness of breath, and headache are more frequently reported by females. A higher rate of nausea and diarrhea may indicate greater gastrointestinal involvement. The intestines may be an active site of viral activity as enterocytes do express ACE2, and stool samples of COVID-19 patients have tested positive for SARS-CoV2 [39]. Meanwhile, other symptoms more likely in females, such as sore throat, runny nose, and headache, are associated with upper respiratory infection and myositis. The difference in immune function may explain these results; a greater antiviral response in various tissues manifests more symptoms [40–42]. Notably, the sex differences in symptom manifestation are only observed in the age group of 20–49, but not 60 or older, suggesting a possible involvement of estrogens. Interestingly, all these female-leaning symptoms, except shortness of breath, are more frequently observed in patients who recovered from COVID-19. Therefore, it is possible that estrogens may also play a role in reducing COVID-19 fatality.

Our observation that there is a correlation between age and hospitalization, ICU admission, recovery time, and fatality is in agreement with previous studies in other countries. Many studies have demonstrated that age is a risk factor for poor COVID-19 outcomes. Analysis of COVID-19 cases in China, the U.K., and Italy consistently demonstrate that increasing age is associated with poorer outcomes [3, 43–45]. For patients younger than 20 years old, we observed a very low number of cases, hospitalization, and ICU admission, and no death. These young patients also had faster recovery and fewer symptoms than older age groups. These findings are also consistent with the current consensus that children and teens are less likely to be infected by SARS-CoV-2 and are more likely to have a mild case if they get infected. For example, a study using data from China, Italy, Japan, Singapore, Canada, and South Korea, found higher symptom rates in older age groups, estimating the rate of clinical presentation of SARS-CoV-2 to range from 20% in children and teenagers to 70% in seniors [46].

## Conclusion

In summary, we demonstrated that females have a lower COVID-19 incidence, hospitalization, ICU admission, and fatality rates in Canada. Our analyses also suggest that estrogens may play a role in reducing COVID-19 incidence and in the development of symptoms, especially those related to increased survival. However, estrogens are unlikely major players in reducing hospital and ICU admission among females. Future studies are warranted to confirm the protective effects of estrogens against SARS-CoV-2 infection.

## Supplementary Information

**Additional file 1: Supplemental Materials and Methods**.

**Additional file 2: Fig. S1. Dataset layout.** Screenshots were taken in MATLAB of the final dataset layout of the Canadian COVID19 dataset (A), Canadian population demographics (B), and Canadian workforce demographics (C). **Fig. S2. Summary of COVID-19 dataset paremeters and potential values. Fig. S3. Age, sex, and symptom segregated COVID-19 cases.** Data represent percentage of cases with symptom relative to total closed cases per sex in each age group. Data were sampled from the ‘A/Symptomatic, Closed cases’ group and analyzed with Fisher’s Exact Test. *p < 0.05, **p < 0.01, ***p < 0.001, >***p < 0.0001.

## Data Availability

The dataset used in this study was obtained from Statistic Canada’s website (https://www150.statcan.gc.ca/t1/tbl1/en/tv.action?pid=1310078101). Source code we developed to analyse the data is available upon request.
