# Correction to: Incidence, clinical features, and outcomes of COVID-19 in Canada: Impact of sex and age

**DOI:** 10.1186/s13048-021-00806-z

**Published:** 2021-04-27

**Authors:** Jacob O’Brien, Kevin Y. Du, Chun Peng

**Affiliations:** 1 Department of Biology, York University, Toronto, Canada; 2 Centre for Research in Biomolecular Interactions, York University, Toronto, ON Canada

**Correction to: J Ovarian Res 13(1), 137 (2020)**

**https://doi.org/10.1186/s13048-020-00734-4**

In the original publication of this article [1], there was an error in Fig. 2i. The error occurred during the final production of the manuscript for publication with a wrong panel appended. Specifically, Fig. 2i should have shown data from patients without the high risk groups; instead, a panel showing data from all patients was included. The corrected Fig. 2 is shown below.

**Fig 2.**
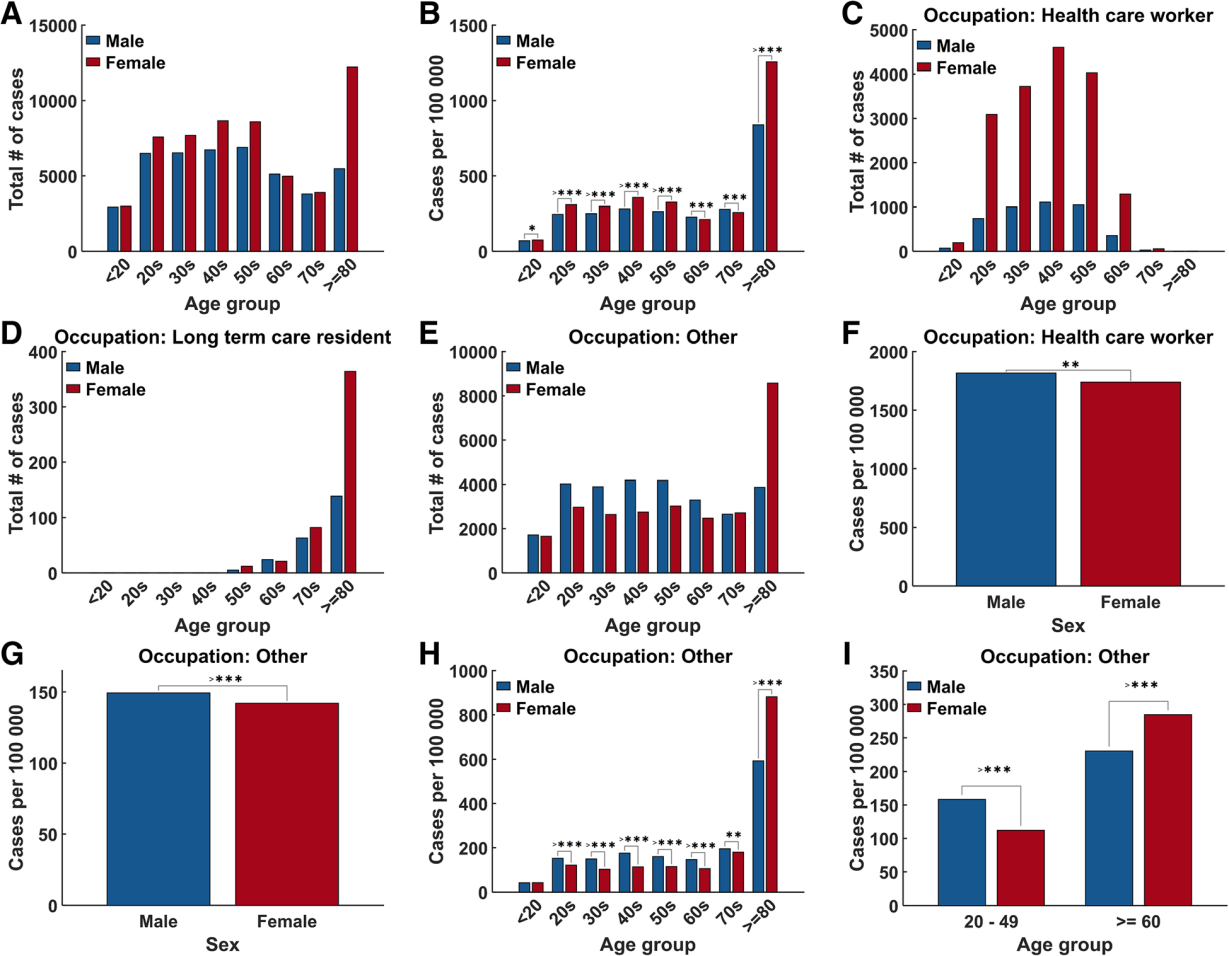
Age, sex, and occupation disaggregated COVID-19 incidence rates. **A** Age/sex distribution of patients from ‘All cases’. **B** Age/sex distribution of patients from ‘All cases’ normalized to population demographics. **C-E** Age distribution of patients with occupation. Occupation “Other” refers to all occupations except health care workers, long-term care residences, and school/daycare workers/attendees. **F** Sex disaggregated Health care worker cases normalized to workforce population demographics (any degree/certificate, all ages, male/female, ‘3 Health occupations’). **G**, **H** Sex and/or age disaggregated ‘Occupation: Other’ cases normalized to Canadian population demographics. **I** Age disaggregated into female reproductive age (20–49) and postmenopausal age (> = 60) for ‘Occupation: Other’ cases. All data was sampled from the ‘All cases’ group. Demographic normalized data was analyzed using *X*^*2*^ goodness-of-fit. *p < 0.05, **p < 0.01, ***p < 0.001, >***p < 0.0001

The original article has been corrected.
