# Supplementary material for: Incidence, clinical features, and outcomes of COVID-19 in Canada: impact of sex and age
Source: J Ovarian Res. 2020 Nov 24;13:137. doi: 10.1186/s13048-020-00734-4 (PMC7684854; doi:10.1186/s13048-020-00734-4)
Supplement: Supplementary file 1 — Additional file 1: Supplemental Materials and Methods. [file 13048_2020_734_MOESM1_ESM.docx]

Incidence, clinical features, and outcomes of COVID-19 in Canada: Impact of sex and age

Jacob O’Brien^a^, Kevin Du^a^, and Chun Peng^a,b*^

^a^Department of Biology, York University, Toronto, Canada

^b^Centre for Research in Biomolecular Interactions, York University, Toronto, ON, Canada

*** Correspondence:**Dr. Chun Peng
[cpeng@yorku.ca](mailto:cpeng@yorku.ca)

**Supplemental Materials and Methods**

**Dataset acquisition and preparation**

All three datasets (COVID19 cases, population demographics, and workplace demographics) were downloaded from Statistics Canada (<https://www.statcan.gc.ca/>). The exact Canadian Workforce Demographic data table downloaded was labaled:

Statistics Canada Catalogue no. 98-400-X2016295. Occupation - National Occupational Classification (NOC) 2016 (693A), Highest Certificate, Diploma or Degree (15), Labour Force Status (3), Age (13A) and Sex (3) for the Labour Force Aged 15 Years and Over in Private Households of Canada, Provinces and Territories, Census Metropolitan Areas and Census Agglomerations, 2016 Census - 25% Sample Data

Each dataset was then prepared within MATLAB by removing unused data to increase readability and reduce processing times. Which columns were removed or how the data were arranged are shown in Suppl. Fig. 1 and under the ‘Datasets’ section within the COVID19.m file. The ‘Dataset’ section has a list of headers before and after removal.

**COVID19.m organization**

The file was organized into sections for easy navigation. The first section, ‘Datasets’, will load all required data and populate variables used to access individual case parameters. The remaining sections were separated by figure. Each figure is self-contained, starting with data queries followed by statistical analysis and then output. For plots, a community designed function, Superbar (<https://www.mathworks.com/matlabcentral/fileexchange/57499-superbar>) was used so that statistical significance markers were also rendered within the plot.
