# Supplementary material for: Incidence, clinical features, and outcomes of COVID-19 in Canada: impact of sex and age
Source: J Ovarian Res. 2020 Nov 24;13:137. doi: 10.1186/s13048-020-00734-4 (PMC7684854; doi:10.1186/s13048-020-00734-4)
Supplement: Supplementary file 2 — Additional file 2: Fig. S1. Dataset layout. Screenshots were taken in MATLAB of the final dataset layout of the Canadian COVID19 dataset (A), Canadian population demographics (B), and Canadian workforce demographics (C). Fig. S2. Summary of COVID-19 dataset paremeters and potential values. Fig. S3. Age, sex, and symptom segregated COVID-19 cases. Data represent percentage of cases with symptom relative to total closed cases per sex in each age group. Data were sampled from the ‘A/Symptomatic, Closed cases’ group and analyzed with Fisher’s Exact Test. *p < 0.05, **p < 0.01, ***p < 0.001, >***p < 0.0001. [file 13048_2020_734_MOESM2_ESM.pdf]

Incidence, clinical features, and outcomes of COVID-19 in Canada: Impact of sex and age

Jacob O'Brien[1], Kevin Du[1], and Chun Peng[1,2*]

[1]Department of Biology, York University, Toronto, Canada

[2]Centre for Research in Biomolecular Interactions, York University, Toronto, ON, Canada

*** Correspondence:**

Dr. Chun Peng

cpeng@yorku.ca

A

CanData

2932509x3 table

	1<br>CaseIdentifierNumber	2<br>CaseInformation	3<br>Value
1	1	'Region'	2
2	1	'Episode_week'	17
3	1	'Episode_week_gro...	0
4	1	'Episode_year'	20
5	1	'Gender'	2
6	1	'Age_group'	8
7	1	'Occupation'	4
8	1	'Asymptomatic'	9
9	1	'Onset_week_of_sy...	99
10	1	'Onset_year_of_sym...	99
11	1	'Symptom_cough'	9
12	1	'Symptom_fever'	9
13	1	'Symptom_chills'	9
14	1	'Symptom_sore_thr...	9
15	1	'Symptom_runny_n...	9

B

CanPop

102x4 table

	1<br>Age	2<br>Total	3<br>Male	4<br>Female
1	-1	37589262	18678085	18911177
2	0	381716	195851	185865
3	1	381655	195516	186139
4	2	386542	198435	188107
5	3	394889	202187	192702
6	4	398373	204180	194193
7	5	401761	205366	196395
8	6	405905	208006	197899
9	7	408230	208483	199747
10	8	407905	208375	199530
11	9	415551	211776	203775
12	10	417389	212892	204497
13	11	417066	212116	204950
14	12	406889	207072	199817
15	13	397226	202088	195138

C

CanWork

405405x13 table

	1<br>DIM_HighestCertificate_Diplon	2<br>MemberID_Hi	3<br>Notes_HighestCerti	4<br>DIM_Age_13A_	5<br>MemberID_Age_	6<br>DIM_Sex_3_	7<br>MemberID_Sex_3_
1	'Total - Highest certificate, d...	1	1	'Total - Age'	1	'Total - Sex'	1
2	'Total - Highest certificate, d...	1	1	'Total - Age'	1	'Total - Sex'	1
3	'Total - Highest certificate, d...	1	1	'Total - Age'	1	'Total - Sex'	1
4	'Total - Highest certificate, d...	1	1	'Total - Age'	1	'Total - Sex'	1
5	'Total - Highest certificate, d...	1	1	'Total - Age'	1	'Total - Sex'	1
6	'Total - Highest certificate, d...	1	1	'Total - Age'	1	'Total - Sex'	1
7	'Total - Highest certificate, d...	1	1	'Total - Age'	1	'Total - Sex'	1
8	'Total - Highest certificate, d...	1	1	'Total - Age'	1	'Total - Sex'	1
9	'Total - Highest certificate, d...	1	1	'Total - Age'	1	'Total - Sex'	1
10	'Total - Highest certificate, d...	1	1	'Total - Age'	1	'Total - Sex'	1
11	'Total - Highest certificate, d...	1	1	'Total - Age'	1	'Total - Sex'	1
12	'Total - Highest certificate, d...	1	1	'Total - Age'	1	'Total - Sex'	1
13	'Total - Highest certificate, d...	1	1	'Total - Age'	1	'Total - Sex'	1
14	'Total - Highest certificate, d...	1	1	'Total - Age'	1	'Total - Sex'	1
15	'Total - Highest certificate, d...	1	1	'Total - Age'	1	'Total - Sex'	1

	8<br>DIM_Occupation_NationalOccupationalCla	9<br>MemberID_Occupatio	10<br>Notes_Occupation_N	11<br>Dim_LabourForceStat	12<br>Dim_LabourForceStatus_3_	13<br>Dim_LabourForceStatus_
1	'Total - Occupation - National Occupati...	1	6	18672470	17230040	1442435
2	'Occupation - Not applicable'	2	7	404350	0	404350
3	'All occupations'	3	8	18268120	17230035	1038090
4	'0 Management occupations'	4	NaN	2013365	1958780	54585
5	'00 Senior management occupations'	5	NaN	225385	221420	3960
6	'001 Legislators and senior management'	6	NaN	225380	221420	3960
7	'0011 Legislators'	7	NaN	7810	7645	170
8	'0012 Senior government managers and ...	8	NaN	17490	17200	285
9	'0013 Senior managers - financial, com...	9	NaN	62610	61440	1170
10	'0014 Senior managers - health, educatio...	10	NaN	26445	25815	625
11	'0015 Senior managers - trade, broadcast...	11	NaN	51375	50505	865
12	'0016 Senior managers - construction, tr...	12	NaN	59655	58810	845
13	'01-05 Specialized middle management ...	13	NaN	650205	632665	17540
14	'011 Administrative services managers'	14	NaN	178340	172830	5515
15	'0111 Financial managers'	15	NaN	74525	72700	1825

Figure S1. Dataset layout. Screenshots were taken in MATLAB of the final dataset layout of the Canadian COVID19 dataset (A), Canadian population demographics (B), and Canadian workforce demographics (C).

Region:

- Atlantic (New Brunswick, Nova Scotia, Prince Edward Island, Newfoundland and Labrador)
- Quebec
- Ontario and Nunavut
- Prairies (Alberta, Saskatchewan, and Manitoba) and the Northwest Territories
- British Columbia and Yukon

Episode week: The episode date is created from the earliest date available from the following series: Symptom Onset Date, Specimen Collection Date, and Laboratory Testing Date.

Sex: Male, Female, Not Stated

Age groups: 0-19, 20-29, 30-39, 40-49, 50-59, 60-69, 70-79, 80+

Occupation:

- Health care worker
- School or daycare worker/attendee
- Long term care resident
- Other
- Not stated

Asymptomatic: Yes, No, Not Stated

Week of symptom onset: 0-56, 99 (Not Stated)

Symptoms: Yes, No, Not Stated

- Cough
- Fever
- Chills
- Sore throat
- Runny nose
- Shortness of breath
- Nausea
- Headache
- Weakness
- Pain
- Irritability
- Diarrhea
- Other

Hospitalization status:

- Hospitalized and in Intensive care unit
- Hospitalized, but not in intensive care unit
- Not hospitalized
- Not Stated/Unknown

Recovered: Yes, No, Not Stated

Recovery week: 0-56, 99 (Not Stated)

Death: Yes, No, Not Stated

Transmission:

- Domestic acquisition - Contact of COVID case, contact with traveller, or unknown source
- International travel
- Not stated

Figure S2. Summary of COVID-19 dataset paremeters and potential values.

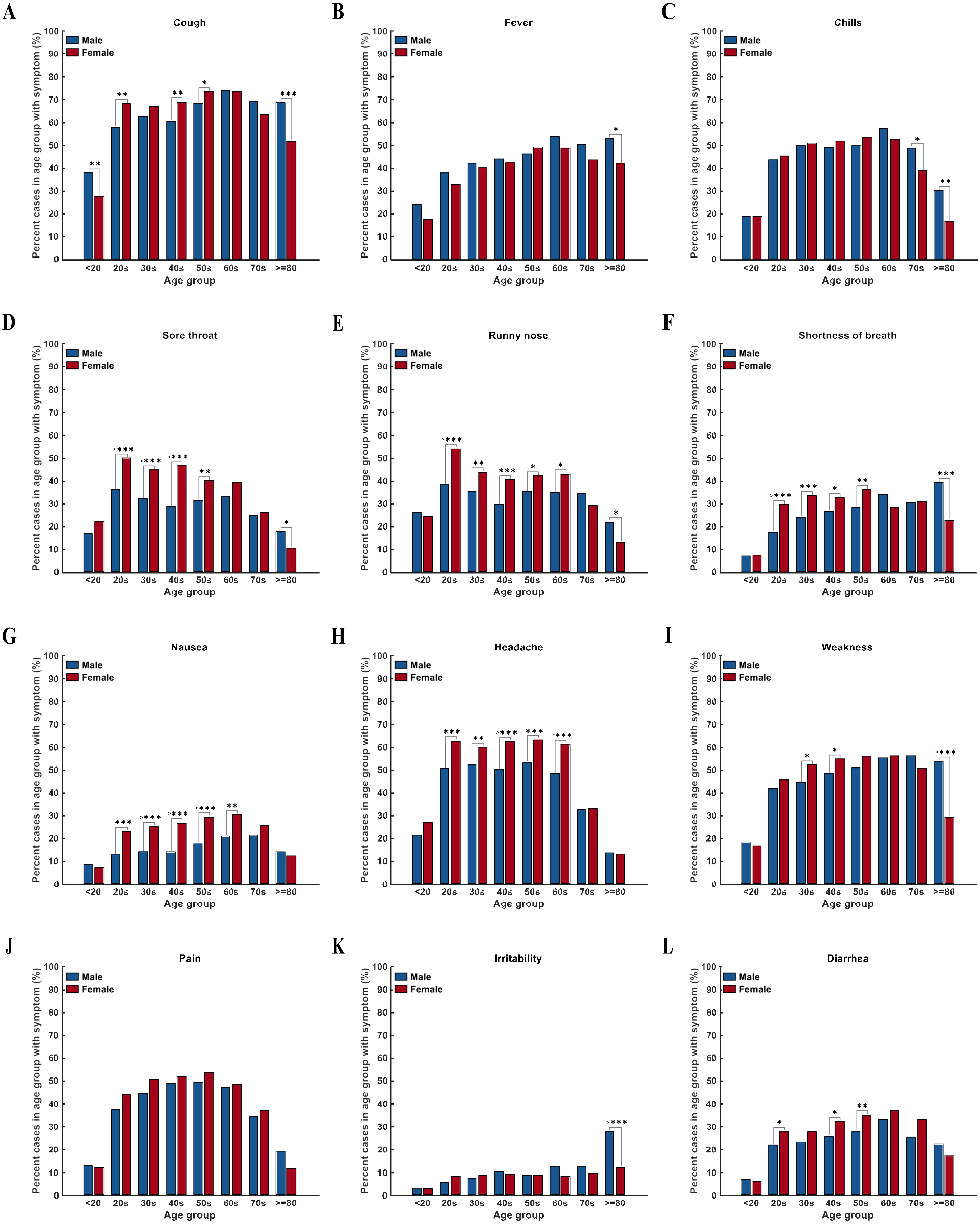

Figure S3. Age, sex, and symptom segregated COVID-19 cases. Data represent percentage of cases with symptom relative to total closed cases per sex in each age group. Data were sampled from the 'A/Symptomatic, Closed cases' group and analyzed with Fisher's Exact Test. *$p < 0.05$, **$p < 0.01$, ***$p < 0.001$, >***$p < 0.0001$.
